# 异基因造血干细胞移植治疗合并肝腺瘤血液病患者8例临床分析

**DOI:** 10.3760/cma.j.cn121090-20240329-00120

**Published:** 2024-09

**Authors:** 云 何, 郑丽 徐, 瑞 马, 静 刘, 园园 张, 萌 吕, 晓冬 莫, 晨华 闫, 于谦 孙, 新羽 张, 昱 王, 晓辉 张, 晓军 黄, 兰平 许

**Affiliations:** 1 北京大学人民医院血液科，北京大学血液病研究所，国家血液系统疾病临床医学研究中心，造血干细胞移植治疗血液病北京市重点实验室，北京 100044 Peking University People's Hospital, Peking University Institute of Hematology, National Clinical Research Center for Hematologic Disease, Beijing Key Laboratory of Hematopoietic Stem Cell Transplantation, Beijing 100044, China; 2 北京大学人民医院影像科，北京 100044 Peking University People's Hospital Department of Medical imaging, Beijing 100044, China

**Keywords:** 腺瘤，肝细胞, 异基因造血干细胞移植, 再生障碍性贫血, 骨髓增生异常综合征, Adenoma, liver cell, Allogeneic hematopoietic stem cell transplantation, Aplastic anemia, Myelodysplastic syndrome

## Abstract

**目的:**

评估异基因造血干细胞移植（allo-HSCT）治疗合并肝腺瘤的血液病患者的安全性。

**方法:**

纳入2010年1月至2024年3月在北京大学人民医院血液科接受allo-HSCT的8例合并肝腺瘤血液病患者，对其临床特征及预后进行回顾性分析。

**结果:**

8例患者中，再生障碍性贫血7例，骨髓增生异常综合征低增生型1例，中位年龄23（13～48）岁，原发病诊断到移植的中位时间为14（6～24）年。服用雄激素到诊断肝腺瘤的中位时间为9（5～13）年。单倍体移植6例，全相合无关供者、全相合同胞供者移植各1例。8例患者中位中性粒细胞植入时间为11.5（11～20）d，中位血小板植入时间为19（10～37）d。7例患者移植后发生巨细胞病毒血症，3例患者发生出血性膀胱炎，2例患者发生急性GVHD。8例患者移植期间及移植后均未出现严重肝功能损害及肝腺瘤破裂出血。4例患者移植后影像学评估肝腺瘤不同程度缩小，另外4例无明显变化。中位随访时间540.5（30～2 989）d，8例中6例存活，2例死亡，死亡与肝腺瘤无直接相关性。

**结论:**

肝腺瘤对血液病患者allo-HSCT的安全性无不良影响。

## Abstract (en)

**Objective:**

To evaluate the safety of patients with hepatic adenoma undergoing allogeneic hematopoietic stem cell transplantation（allo-HSCT）.

**Methods:**

A retrospective analysis of the clinical characteristics and prognosis of eight patients with hepatic adenoma who underwent allo-HSCT in the Hematology Department of Peking University People's Hospital from January 2010 to March 2024 was conducted.

**Results:**

Of the eight patients who underwent allo-HSCT with hepatic adenoma, one patient was considered MDS-h transfusion-dependent and seven had aplastic anemia. The median age of the patients was 23 years（13–48 years）. The median time from the diagnosis of AA or MDS to transplantation was 14 years（6–24 years）, whereas the median time from taking androgens to diagnosing hepatic adenoma was 9 years（5–13 years）. Six cases underwent haplo-HSCT, one case underwent matched unrelated donor HSCT, and one case underwent matched related donor HSCT. All patients achieved neutrophil engraftment at a median time of 11.5 days（11–20 days）and PLT engraftment within 60 days at a median of 19 days（10–37 days）after haplo-HSCT. Moreover, seven patients developed CMV anemia after transplantation, three patients had hemorrhagic cystitis, and two patients developed acute GVHD. During and after transplantation, eight patients did not show severe liver function damage or rupture of hepatic adenoma. In relation to imaging size, four patients showed varying degrees of reduction in hepatic adenoma size after transplantation, whereas four patients did not show significant changes in hepatic adenoma size after transplantation. The median follow-up time was 540.5（30–2 989）days. Of the eight patients, six survived and two died. Furthermore, no direct correlation was observed between death and hepatic adenoma.

**Conclusion:**

Patients with hepatic adenomas undergoing allo-HSCT are not contraindications for transplantation, which will not increase transplant-related mortality.

再生障碍性贫血（aplastic anemia, AA）是一种获得性骨髓衰竭综合征，以全血细胞减少及其所致贫血、感染、出血为主要表现，国内流行病学调查发病率为0.74/10万[1]。对于重型AA（SAA）、极重型AA（VSAA）及输血依赖的非重型AA（NSAA），近年来异基因造血干细胞移植（allo-HSCT）治疗取得显著进展，极大地改善了患者预后。对于NSAA，雄激素联合/不联合免疫抑制剂是最常见的治疗办法，雄激素总体有效率为27％～50％，其作用机制包括提高体内红细胞生成素水平、直接促进红系造血和端粒调节作用，一般需用药6个月才能判断疗效，且部分患者有药物依赖性需要持续治疗，目前常用的如十一酸睾酮和司坦唑醇等[2]–[4]。骨髓增生异常综合征（MDS）是起源于造血干细胞的克隆性疾病，治疗方案需根据预后分组、年龄等进行个体化制定方案，雄激素是部分低危MDS促造血治疗的常见用药。长期应用雄激素或合成代谢性类固醇（AAS）可导致肝腺瘤[5]–[12]。肝腺瘤在长期服用避孕药女性群体中的发病率为（3～4）/10万，而在不服用避孕药及服药史短于2年的女性群体中的发病率仅为0.1/10万[13]–[14]。肝腺瘤的临床表现与肿瘤大小、部位及有无并发症有关。5％～10％的患者无任何症状，约1/3的患者有腹部包块、右上腹痛、恶心、纳差等不适；当肿瘤发生破裂出血时，可有急腹症表现；还可能恶变为肝细胞肝癌[15]–[16]。

allo-HSCT是AA及MDS最有效的治疗方法[17]–[19]。合并肝腺瘤对血液病患者行allo-HSCT的影响尚无相关报道。本研究对在本中心接受allo-HSCT的8例合并肝腺瘤血液病患者进行回顾性分析，旨在探究合并肝腺瘤对血液病患者allo-HSCT的安全性是否构成影响。

## 病例与方法

一、病例

本项回顾性研究纳入2010年1月至2024年3月在北京大学人民医院血液科合并肝腺瘤并接受allo-HSCT的8例血液科患者。以检索病历方式获取患者年龄、性别、供受者性别异同、疾病诊断、人类白细胞抗原（HLA）配型、干细胞来源、预处理方案、移植物抗宿主病（GVHD）发生、粒细胞及血小板植入时间、移植前后肝腺瘤的大小、移植前后肝功能等临床信息，分析合并肝腺瘤患者的移植预后。

二、移植方案

1. 预处理：单倍体移植AA患者：白消安（Bu）3.2 mg·kg^−1^·d^−1^，−8 d～−7 d；环磷酰胺（Cy）50 mg·kg^−1^·d^−1^，−5 d～−2 d；兔抗人胸腺细胞免疫球蛋白（rATG）2.5 mg·kg^−1^·d^−1^，−5 d～−2 d。心脏毒性高危SAA患者：Bu 3.2 mg·kg^−1^·d^−1^，−8 d、−7 d；Cy 25 mg·kg^−1^·d^−1^，−5 d～−2 d；氟达拉滨（Flu）30 mg·m^−2^·d^−1^，−6 d～−2 d；rATG 2.5 mg·kg^−1^·d^−1^，−5 d～−2 d。全相合无关/同胞供者移植AA患者：Cy 50 mg·kg^−1^·d^−1^，−5 d～−2 d；rATG 2.5 mg·kg^−1^·d^−1^，−5 d～−2 d。

单倍体移植MDS患者：阿糖胞苷（Ara-C）4 g/m^2^，−9 d；Bu 3.2 mg·kg^−1^·d^−1^，−8 d～−6 d；Cy 1.8 g·m^−2^·d^−1^，−5 d～−4 d；司莫司汀（MeCCNU）250 mg/m^2^，−3 d；rATG 2.5 mg·kg^−1^·d^−1^，−5 d～−2 d。

2. GVHD防治：采用环孢素A+短程甲氨蝶呤+霉酚酸酯方案。

三、定义

连续3 d外周血中性粒细胞计数（ANC）>0.5×10^9^/L定义为粒细胞植入，ANC>0.5×10^9^/L的第1天定义为粒细胞植入时间。连续7 d外周血PLT>20×10^9^/L且脱离血小板输注定义为血小板植入，PLT>20×10^9^/L的第1天为血小板植入时间。

四、肝腺瘤的诊断

肝腺瘤的诊断目前是临床结合影像学和（或）病理活检，病理是诊断肝腺瘤的金标准[14]，MRI对肝腺瘤的诊断也有较高的准确率[20]–[21]。本组患者血象均较差，病理活检标本难以获得，除例5移植前病理活检证实为肝腺瘤（该患者活检后出现肝腺瘤出血行肝脏动脉血管栓塞术），其余7例患者均根据病史及MRI结果经多学科会诊而确定诊断。

五、随访

以检索病历方式获取随访数据，随访截止时间为2023年12月31日。总生存（OS）时间是指造血干细胞回输至随访截止或死亡的时间。

## 结果

一、患者一般情况

8例患者中，AA 7例，MDS低增生型（MDS-h）1例，男4例，女4例，中位年龄为23（13～48）岁，诊断AA或MDS到移植的中位时间为14（6～24）年，服用雄激素到诊断为肝腺瘤的中位时间为9（5～13）年。详见表1。

**表1:** 8例合并肝腺瘤血液病患者的一般情况及异基因造血干细胞移植相关资料

例号	性别	年龄(岁)	诊断	诊断到移植时间（年）	服用雄激素到肝腺瘤诊断时间（年）	供者	供/受者血型	预处理	MNC输注量（×10^8^/kg）	CD34^+^细胞输注量（×10^6^/kg）	粒细胞植入（d）	血小板植入（d）	移植后合并症
1	女	22	SAA	16	13	单倍体（父）	B+/O+	BuCy+rATG	8.53	3.32	11	37	CMV血症
2	女	25	SAA	15	7.5	单倍体（父）	A+/O+	BuCy+Flu+rATG	11.23	3.96	20	−	CMV血症，出血性膀胱炎Ⅲ度，植入功能不良，感染性休克
3	男	22	NSAA输血依赖	6	5	单倍体（父）	O+/B+	BuCy+Flu+rATG	11.2	8.3	11	18	CMV血症
4	男	29	NSAA输血依赖	24	9	单倍体（父）	B+/AB+	BuCy+Flu+rATG	11.89	5.23	11	22	急性GVHD Ⅱ度，CMV血症，新型冠状病毒肺炎
5	女	23	MDS-h输血依赖	8	8	单倍体（父）	B+/B+	BuCy+rATG	8.65	4.79	11	20	植入综合征，CMV血症
6	女	23	SAA	13	13	全相合无关	O+/A+	Cy+rATG	9	2.79	19	−	急性GVHD，CMV血症，中枢神经系统感染
7	男	48	SAA	20	10	全相合同胞	A+/B+	BuCy+Flu+rATG	13.01	2.68	15	15	CMV血症，EBV血症，出血性膀胱炎Ⅰ度
8	男	13	NSAA输血依赖	11	9	单倍体（父）	AB+/A+	BuCy+Flu+rATG	7.38	2.53	12	10	出血性膀胱炎Ⅱ度

**注** SAA：重型再生障碍性贫血；NSAA：非重型再生障碍性贫血；MDS-h：骨髓增生异常综合征低增生型；Bu：白消安；Flu：氟达拉滨；Cy：环磷酰胺；rATG：兔抗人胸腺细胞免疫球蛋白；MNC：单个核细胞；CMV：巨细胞病毒；EBV：EB病毒；GVHD：移植物抗宿主病

二、移植相关情况

单倍体移植6例，全相合无关供者、全相合同胞供者移植各1例。MDS患者移植物为外周血干细胞，其余7例AA患者移植物均为骨髓+外周血干细胞。单个核细胞中位输注量为10.10（7.38～13.01）×10^8^/kg，CD34^+^细胞中位输注量为3.64（2.53～8.30）×10^6^/kg。8例患者中位中性粒细胞植入时间为11.5（11～20）d，中位血小板植入时间为19（10～37）d。7例患者移植后发生巨细胞病毒血症，3例患者发生出血性膀胱炎，2例患者发生急性GVHD（表1）。

8例患者移植过程及移植后有6例出现轻度肝功能不全（表现为转氨酶及胆红素升高，均考虑为药物性肝损害，予保肝治疗后好转）。8例患者均未出现移植后肝脏相关严重不良事件（肝窦静脉闭塞综合征、严重肝功能不全等），详见表2。

**表2:** 8例合并肝腺瘤血液病患者异基因造血干细胞移植前后肝腺瘤情况及随访结果

例号	移植前肝腺瘤情况	移植后ALT峰值（U/L）	移植后AST峰值（U/L）	移植后TBIL峰值（µmol/L）	移植后肝腺瘤变化	移植后总生存时间（d）
1	多发，最大3.7 cm×2.5 cm	359	156	26	1.5年评估多发，部分病灶消失，余较前缩小（最大2.1 cm×1.9 cm）	2 989
2	多发，最大2.2 cm×1.6 cm	397	530	75	2个月评估多发，最大2.0 cm×1.6 cm	88 （死于感染中毒性休克）
3	多发，最大4.9 cm×3.1 cm；停雄激素1年最大2.0 cm×1.6 cm	253	85	18	1个月评估多发，最大1.5 cm×1.3 cm	462
4	多发，最大5.3 cm×3.7 cm	372	151	42	8个月评估多发，最大1.7 cm×1.2 cm	599
5	多发，最大7.2 cm×4.9 cm	103	94	16	5个月评估多发，最大5.6 cm×3.0 cm	727
6	单发3.8 cm×2.3 cm	350	114	24	1年评估单发，3.8 cm×2.1 cm	482 （死于中枢神经系统感染）
7	多发，最大3.0 cm×2.7 cm	99	51	15	4.5年评估多发，最大2.8 cm×2.5 cm	1 529
8	多发，最大3.7 cm×3.1 cm	277	187	36	1个月评估多发，最大3.3 cm×2.9 cm	30

**注** SAA：重型再生障碍性贫血；NSAA：非重型再生障碍性贫血；MDS-h：骨髓增生异常综合征低增生型；TBIL：总胆红素；ALT：丙氨酸转氨酶；AST：天氡氨酸转氨酶

三、肝腺瘤情况

8例患者有7例为多发病灶，1例为单发病灶。8例患者移植后均未发生肝腺瘤破裂出血。4例患者移植后影像学检查显示肝腺瘤不同程度缩小，另外4例患者无明显变化（表2）。

四、随访结果

8例患者中位随访时间为540.5（30～2 989）d，6例存活，2例死亡（例2移植后植入功能不良，+88 d死于感染中毒性休克；例6移植后482 d发生中枢神经系统感染持续癫痫）。未发生肝腺瘤相关死亡。

## 讨论

allo-HSCT是SAA、输血依赖的NSAA以及MDS的主要治疗手段。随着移植技术的进步，越来越多病史长、一般情况较差的AA/MDS的患者有了移植机会。本研究中的8例患者病史均较长，诊断到移植的中位时间为14（6～24）年，服用雄激素的中位时间为9（5～13）年。8例患者移植过程相关安全，移植期间及移植后无肝腺瘤破裂、出血表现，所有患者肝腺瘤稳定或较移植前明显缩小。

肝腺瘤是罕见的肝脏良性肿瘤，据报道长期服用避孕药者该病的发病率为（3～4）/10万，活检证实的发病率为0.07/10万[22]。病理是肝腺瘤的诊断金标准，但经皮穿刺活检可导致出血、肿瘤转移等不良后果，且活检病理阴性并不能排除恶性肿瘤，只有当影像学检查不能确诊且活检可能会带来治疗管理上的改变时，才应该考虑活检[14]。既往肝脏肿瘤指南推荐怀疑肝腺瘤患者进行增强MRI明确肝腺瘤亚型的诊断率高达80％[23]–[24]。本组8例患者有1例行经皮穿刺活检并确诊，但术后出现病灶出血增大、被迫行肝血管栓塞止血，余7例患者均通过临床病史、增强MRI和多学科会诊而明确诊断。

肝腺瘤的治疗目前无标准推荐，是否选择手术治疗尚有争议。文献[14],[25]推荐：<5 cm的腺瘤以及AAS停药后消退的腺瘤，可以保守治疗；建议所有患者停止使用外源性雄激素，<3.5 cm的腺瘤和随着时间推移体积缩小的肿瘤很少发生恶变；腺瘤>5 cm、甲胎蛋白（AFP）及β-catenin升高的男性考虑手术。有文献总结了11例AAS相关肝腺瘤患者，发现服用AAS到肝腺瘤的中位时间是13（0.5～20）年[11]，本组8例患者服用雄激素到诊断肝腺瘤的中位时间为9（5～13）年。在既往文献报道中，有6例患者采用单纯停用AAS治疗AAS相关肝腺瘤[9],[11],[26]–[27]，其中遗传性血管性水肿3例、AA 2例、雄激素过度使用1例，这6例患者中2例停药2年无变化，2例分别在停药18、26个月后病灶消失，另外2例病灶缩小。本组8例患者因移植前血象极差、手术风险大，均在移植前后停用雄激素，移植后4例患者肝腺瘤不同程度缩小，余4例患者无明显变化，提示对于合并雄激素类药物所致肝腺瘤的血液系统疾病患者，选择停药观察可能更为恰当。

综上所述，本组8例患者在移植前预处理和移植后均未发生严重肝功能损害及肝腺瘤破裂出血，也未发生肝腺瘤相关死亡，初步表明肝腺瘤对血液病患者allo-HSCT的安全性无不良影响。本文为回顾性研究且例数较少，以上结论尚需大样本及前瞻性研究加以验证。
